# Cytotoxicity and Structure-Activity Relationships of Xanthone Derivatives from *Mesua beccariana*, *Mesua ferrea* and *Mesua congestiflora* towards Nine Human Cancer Cell Lines

**DOI:** 10.3390/molecules18021985

**Published:** 2013-02-04

**Authors:** Soek Sin Teh, Gwendoline Cheng Lian Ee, Siau Hui Mah, Yang Mooi Lim, Zuraini Ahmad

**Affiliations:** 1 Department of Chemistry, Faculty of Science, Universiti Putra Malaysia, Serdang 43400, Selangor, Malaysia; 2 Faculty of Medicine and Health Science, Universiti Tunku Abdul Rahman, Kajang 43000, Selangor, Malaysia; 3 Department of Biomedical Science, Faculty of Medicine and Health Sciences, Universiti Putra Malaysia, Serdang 43400, Selangor, Malaysia

**Keywords:** cytotoxicity, *Mesua beccariana*, *Mesua ferrea*, *Mesua congestiflora*, structure-activity relationship, xanthones

## Abstract

The cytotoxic structure-activity relationships among a series of xanthone derivatives from *Mesua beccariana*, *Mesua ferrea* and *Mesua congestiflora* were studied. Eleven xanthone derivatives identified as mesuarianone (**1**), mesuasinone (**2**), mesuaferrin A (**3**), mesuaferrin B (**4**), mesuaferrin C (**5**), 6-deoxyjacareubin (**6**), caloxanthone C (**7**), macluraxanthone (**8**), 1,5-dihydroxyxanthone (**9**), tovopyrifolin C (**10**) and *α*-mangostin (**11**) were isolated from the three *Mesua* species. The human cancer cell lines tested were Raji, SNU-1, K562, LS-174T, SK-MEL-28, IMR-32, HeLa, Hep G2 and NCI-H23. Mesuaferrin A (**3**), macluraxanthone (**8**) and *α*-mangostin (**11**) showed strong cytotoxicities as they possess significant inhibitory effects against all the cell lines. The structure-activity relationship (SAR) study revealed that the diprenyl, dipyrano and prenylated pyrano substituent groups of the xanthone derivatives contributed towards the cytotoxicities.

## 1. Introduction

**Figure 1:**
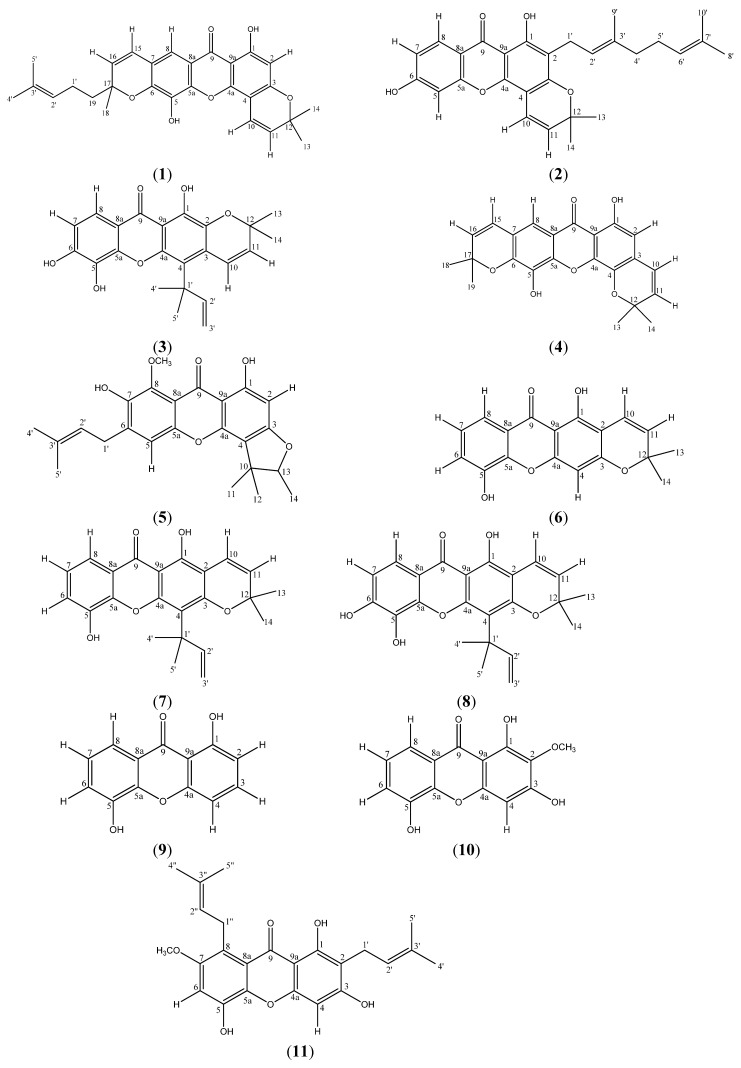
Structures of compounds **1**–**11**.

The genus *Mesua*, from the Clusiaceae family was selected for preliminary pharmacognosy analysis as some previous chemical investigations on this genus have revealed their activities to be due to the phloroglucinols, xanthones and neoflavonoids present in these plants [1,2,3]. 6-Deoxyjacareubin (**6**) has been reported for its growth inhibitory effect against HL-60 (leukemia) cells [4], antioxidant activity [5] as well as platelet-activating factor receptor binding inhibitory effects [6,7]. There are also reports on caloxanthone C (**7**) and macluraxanthone (**8**) having good activities on *Plasmodium falciparum* [8]. Macluraxanthone (**8**) has also been reported to exhibit potent cholinesterase inhibitory [9], strong antioxidant and cytotoxic activities [10]. On the other hand, 1,5-dihydroxyxanthone (**9**) has been shown to display moderate cytotoxicity towards KB cells [11]. *α*-Mangostin (**11**) however, showed anti-proliferative and pro-apoptotic effects on chronic B leukemia cells [12] and was cytotoxic on canine osteosarcoma D-17 cells [13]. The biological activities of compounds **1**–**5** and **10** have not been reported. We report here the biological activities for compounds **1**–**11** (Figure 1) as well as the SAR for these compounds.

## 2. Results and Discussion

Cytotoxicities of all the isolated xanthone derivatives were evaluated against a panel of cancer cell lines which included Raji (human B lymphocyte), SNU-1 (human gastric carcinoma), K562 (human erythroleukemia cells), LS-174T (human colorectal adenocarcinoma), HeLa (human cervical cells), SK-MEL-28 (human malignant melanoma cells), NCI-H23 (human lung adenocarcinoma), IMR-32 (human neuroblastoma) and Hep G2 (human hepatocellular liver carcinoma). All the xanthone derivatives isolated from *M. beccariana*, *M. ferrea* and *M. congestiflora* are listed in Table 1. The IC_50_ values of tested cancer cells treated with the isolated xanthone derivatives are summarized in Table 2. Quercetin was used as a standard drug throughout the experiments. The structure-activity relationships among this series of xanthone derivatives (Table 1) were then comparatively predicted for all the investigated cell lines.

Xanthones with a prenyl group such as mesuaferrin A (**3**), caloxanthone C (**7**) and macluraxanthone (**8**), or diprenylated like *α*-mangostin (**11**) or geranylated as in mesuasinone (**2**) and have a fused pyrano ring showed prominent inhibitory effects towards Raji cells. Interestingly, the hydroxylation of the xanthone nucleus at C-6 of mesuaferrin A (**3**) and macluraxanthone (**8**) led to a significant increase in the cytotoxic effect, as shown in Table 1. The decrease in cytotoxic effect of mesuarianone (**1**) was probably due to the indirectly attached prenyl group at the pyrano moiety. Xanthone derivatives such as 6-deoxyjacareubin (**6**) and mesuaferrin B (**4**) with no prenyl chain and mesuaferrin C (**5**) with a furano ring have their cytotoxic effects further reduced. On the other hand, the simple xanthone, 1,5-dihydroxyxanthone (**9**) as well as tovopyrifolin C (**10**) which is methoxylated at C-2 of the xanthone nucleus did not inhibit the growth of the Raji cells or any of the other cancer cell lines tested.

**Table 1:** Xanthone derivatives from *M. beccariana*, *M. ferrea* and *M. congestiflora*.

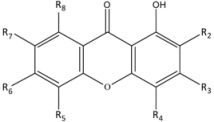							
Compds.	R_2_	R_3_	R_4_	R_5_	R_6_	R_7_	R_8_
**1**	H	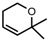		OH	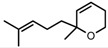		H
**2**	geranyl	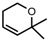		H	OH	H	H
**3**	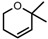		prenyl B	OH	OH	H	H
**4**	H	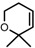		OH	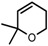		H
**5**	H	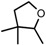		H	prenyl A	OH	OCH_3_
**6**	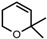		H	OH	H	H	H
**7**	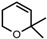		prenyl B	OH	H	H	H
**8**	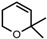		prenyl B	OH	OH	H	H
**9**	H	H	H	OH	H	H	H
**10**	OCH_3_	OH	H	OH	H	H	H
**11**	prenyl A	OH	H	OH	H	OCH_3_	prenyl A

prenyl A: 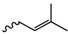; prenyl B: 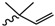; geranyl: 
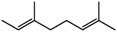
.

**Figure 2:**
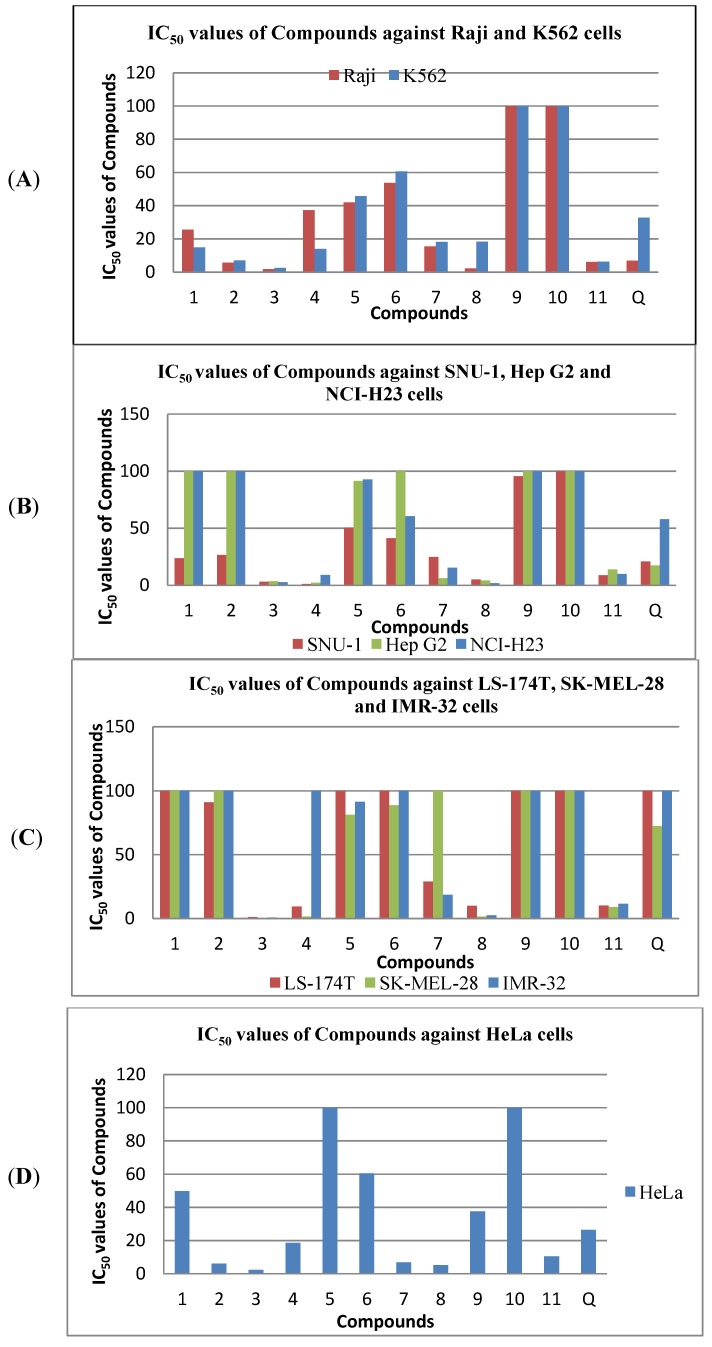
IC_50_ values of Compounds **1**–**11** and quercetin against (**A**) Raji and K562 cells, (**B**) SNU-1, Hep G2 and NCI-H23 cells, (**C**) LS-174T, SK-MEL-28 and IMR-32 cells and (**D**) HeLa cells.

Differences in the activity of compounds **1**, **4** and **8** towards K562 and Raji cells were observed. (Table 2). Apparently, the prenylated methyl groups of the pyrano moiety in mesuarianone (**1**) and the dipyrano group in mesuaferrin B (**4**) induce inhibitory activity towards K562 cells. Moreover, the presence of the prenylated pyrano with an oxygen atom at R_3_, as in macluraxanthone (**8**) (Table 1) reduces the activity towards the K562 cell line (Figure 2A).

**Table 2:** IC_50_ values of a Panel of Human Cancer Cell Lines Treated with Pure Compounds from the three *Mesua* species.

Compds.	Cell Lines with IC_50_ values (µM)								
	Raji	SNU-1	K562	LS-174T	SK-MEL-28	IMR-32	HeLa	Hep G2	NCI-H23
**1**	25.46 ± 0.90	23.78 ± 1.33	14.93 ± 0.29	-	-	-	49.83 ± 1.31	-	-
**2**	5.61 ± 2.25	26.61 ± 0.58	7.02 ± 0.50	91.19 ± 1.45	-	-	6.12 ± 1.82	-	-
**3**	1.78 ± 0.57	3.17 ± 1.05	2.54 ± 2.00	1.17 ± 2.02	0.36 ± 2.38	0.79 ± 1.24	2.39 ± 1.07	3.68 ± 2.41	2.64 ± 2.95
**4**	37.24 ± 0.72	1.19 ± 1.36	13.95 ± 0.72	9.31 ± 2.36	1.48 ± 1.24	-	18.60 ± 1.52	2.32 ± 1.07	8.98 ± 1.32
**5**	41.90 ± 1.51	50.80 ± 1.50	45.73 ± 1.39	-	81.29 ± 2.30	91.46 ± 1.45	-	91.46 ± 1.04	92.68 ± 1.25
**6**	53.77 ± 1.11	41.29 ± 1.36	60.65 ± 0.64	-	88.71 ± 2.19	-	60.48 ± 1.59	-	60.48 ± 1.87
**7**	15.50 ± 1.99	24.79 ± 1.96	18.20 ± 1.32	28.94 ± 2.20	-	18.60 ± 1.03	6.88 ± 1.54	6.22 ± 1.49	15.42 ± 2.88
**8**	2.18 ± 1.87	5.08 ± 2.20	18.25 ± 1.25	9.90 ± 2.47	1.40 ± 1.69	2.49 ± 0.54	5.28 ± 2.04	4.24 ± 2.13	1.85 ± 1.76
**9**	-	95.53 ± 1.81	-	-	-	-	37.68 ± 2.53	-	-
**10**	-	-	-	-	-	-	-	-	-
**11**	6.10 ± 1.20	8.90 ± 1.06	6.34 ± 2.24	10.17 ± 1.68	8.88 ± 1.77	11.44 ± 2.45	10.49 ± 2.05	13.90 ± 1.24	10.00 ± 0.53
**Q**	6.89 ± 0.80	20.86 ± 1.93	32.75 ± 3.20	-	72.45 ± 2.07	-	26.49 ± 1.74	17.25 ± 1.85	57.95 ± 1.88

IC_50_ values less than 10 µM indicates strong activity; 10 µM < IC_50_ values < 50 µM indicates moderate activity; (-): IC_50_ values more than 50 µM indicates weak activity. Each data represents the mean of three independent experiments; Q represents Quercetin.

For SNU-1, Hep G2 and NCI-H23 cell lines, significant activities were noted with the attachment of diprenyls to the xanthone nucleus as in *α*-mangostin (**11**), dipyrano rings as in mesuaferrin B (**4**), and for prenylated tri-hydroxylated xanthones with a pyrano ring like mesuaferrin A (**3**) and macluraxanthone (**8**). The prenylated dihydroxylated monopyranoxanthone, caloxanthone C (**7**) showed a significant cytotoxicity towards Hep G2. However, the cytotoxicity towards SNU-1 cells was reduced due to the absence of one OH group. The methoxylated diprenylated xanthone derivative, *α*-mangostin (**11**) revealed significant anti-proliferative effects against both SNU-1 and NCI-H23 while the inhibitory effect towards Hep G2 was reduced due to the presence of its diprenyl groups. On the other hand, derivatives having a fused furano ring, mesuaferrin C (**5**), indirectly prenylated dipyrano ring, mesuarianone (**1**), monopyrano ring, 6-deoxyjacareubin (**6**), geranyl group with pyrano ring, mesuasinone (**2**), as well as dihydroxylated xanthones, 1,5-dihydroxyxanthone (**9**) have their cytotoxicities towards SNU-1 cells reduced. Meanwhile, the monopyrano derivative, 6-deoxyjacareubin (**6**) and furano derivative, mesuaferrin C (**5**) have reduced inhibitory effects against NCI-H23 cells. The latter furano derivative also possesses weak cytotoxicity towards Hep G2 cells. Both NCI-H23 and Hep G2 cells were less vulnerable to the rest of the xanthone derivatives (Figure 2B).

Mesuaferrin A (**3**), macluraxanthone (**8**), *α*-mangostin (**11**) and mesuaferrin B (**4**) demonstrated significant cytotoxic activity towards LS-174T cells. This could be due to the prenylated pyrano moiety together with dihydroxylation at C-5 and C-6 or the presence of diprenyl or dipyrano moieties. However, caloxanthone C (**7**) and mesuasinone (**2**) which are prenylated or geranylated dihydroxy pyranoxanthone derivatives showed a decrease in inhibitory activity against the LS-174T cells. 6-Deoxyjacareubin (**6**), with a fused monopyrano ring and mesuaferrin C (**5**) with a fused furano ring, had their inhibitory activity towards LS-174T cell line diminished. The non-prenylated 1,5-dihydroxyxanthone (**9**) along with mesuarianone (**1**) with a prenylated methyl group at the pyrano ring also had no inhibitory activity towards LS-174T cell line (Figure 2C).

The SK-MEL-28 cell growth was highly suppressed by the same group of xanthone derivatives as described for LS-174T cells. However, the cytotoxicity was increased by the occurrence of a monopyrano ring in 6-deoxyjacareubin (**6**) and a furano ring in mesuaferrin C (**5**) when compared to the LS-174T cells. The replacement of OH by H in caloxanthone C (**7**) as compared in macluraxanthone (**8**) astonishingly reduces the activity of the xanthone. On the other hand, the indirectly-attached prenylated dipyrano ring in mesuarianone (**1**), the geranylated pyrano ring in mesuasinone (**2**) and the dihydroxyl group in 1,5-dihydroxyxanthone (**9**) were inactive towards SK-MEL-28 cells (Figure 2C).

The difference in the structure-activity relationship between SK-MEL-28 and IMR-32 cell lines was noticed for mesuaferrin B (**4**) and caloxanthone C (**7**). The inhibitory potency of the dipyrano derivative, mesuaferrin B (**4**) towards IMR-32 cells remarkably decline compared to SK-MEL-28 while the absence of the OH group at C-6 of caloxanthone C (**7**) was associated with a higher cytotoxicity compared to that on SK-MEL-28 cells (Figure 2C).

All the xanthone derivatives revealed significant cytotoxic activities towards HeLa cells except for the indirectly prenylated dipyrano-mesuarianone (**1**), dipyrano-mesuaferrin B (**4**), monopyrano-6-deoxyjacareubin (**6**), dihydroxylated 1,5-dihydroxyxanthone (**9**) and diprenylated *α*-mangostin (**11**) which did not show significant inhibitory growth of the cells. Meanwhile, the presence of a furano moiety in mesuaferrin C (**5**) further diminished remarkably its inhibitory activity towards HeLa cells (Figure 2D).

## 3. Experimental

### 3.1. General

All the apparatus used for cell culture were sterilized and decontaminated using a Hirayama HICLAVE HVE-50 (UTAR, Kuala Lumpur, Malaysia). Cell culture handling was carried out in an ESCO Class II BSC Biosafety Cabinet (UTAR, Kuala Lumpur, Malaysia). The healthy cells were spun down, adherent together and separated from unhealthy and dead cells by using Thermo Scientific Sorvall ST 16R centrifuge (UTAR, Kuala Lumpur, Malaysia). All cultures were incubated in 5% CO_2_ humidified incubator at 37 °C (ESCO Celculture CO_2_ Incubator model number CCL-170B-8). Cell stocks were placed in a −86 °C ultra- low temperature freezer (Scancool SCL 50 P, UTAR, Kuala Lumpur, Malaysia) and preserved in a liquid nitrogen tank (Taylor-Wharton LS300, UTAR, Kuala Lumpur, Malaysia).

### 3.2. Plant Material

The stem bark of *M. beccariana*, root bark of *M. ferrea* and roots of *M. congestiflora* were collected from the Sri Aman district in Sarawak, Malaysia. All the plant materials were identified by Associate Professor Dr. Rusea Go, Department of Biology, Faculty of Science, Universiti Putra Malaysia and deposited in the herbarium of the Department of Biology, Faculty of Science, Universiti Putra Malaysia.

### 3.3. Extraction and Isolation

The air-dried and powdered *M. beccariana* stem bark (3 Kg) was extracted successively with *n*-hexane, dichloromethane, ethyl acetate and methanol (2 L, 5 times, 22 °C). The extracts were dried under reduced pressure using a rotary evaporator to obtain the hexane (15.6 g), dichloromethane (21.2 g), ethyl acetate (15.8 g) and methanol (80.5 g) extracts, respectively. Column chromatographic purification (Merck silica gel, hexane, dichloromethane, ethyl acetate and methanol) of the hexane extract gave two xanthones, mesuarianone (**1**) and mesuasinone (**2**) [14]. Meanwhile, the ethyl acetate extract gave a xanthone 6-deoxyjacareubin (**6**) [15]. The same extraction procedure which was carried out for *M. ferrea* afforded hexane (49.6 g), dichloromethane (19.5 g), ethyl acetate (16.7 g) and methanol (62.2 g) extracts. Column chromatography purification of the hexane extract yielded four compounds which were identified as mesuaferrin A (**3**) [16], mesuaferrin C (**5**) [17], caloxanthone C (**7**) [15] and macluraxanthone (**8**) [18]. Meanwhile, the dichloromethane extract gave mesuaferrin B (**4**) [16] while the ethyl acetate extract furnished two xanthones which were 1,5-dihydroxyxanthone (**9**) [19] and tovopyrifolin C (**10**) [20]. Furthermore, the 0.84 Kg air-dried and milled sample of *M. congestiflora* roots were macerated consecutively with hexane, ethyl acetate and methanol at room temperature. The extracts were desiccated under reduced pressure using a rotary evaporator to yield hexane (5.5 g), ethyl acetate (61.0 g) and methanol (120.5 g) extracts. Column chromatographic separation of the hexane extract afforded a xanthone *α*-mangostin (**11**) [21] (Figure 1). Compounds were identified using NMR, GCMS and FTIR. Spectral data for compounds **1**–**11 **are in agreement with literature data [14,15,16,17,18,19,20,21].

### 3.4. Cytotoxicity Assay

The cytotoxic assays were performed by the MTT assay as described by Mosmann [22]. In this study, nine cancer cell lines obtained from Universiti Tunku Abdul Rahman (UTAR) were used: Raji, SNU-1, K562, LS-174T, SK-MEL-28, IMR-32, HeLa, Hep G2 and NCI-H23. Quercetin, which was used as a standard, showed cytotoxic activity with IC_50_ values ranging from 6.89–72.45 µM except for LS-174T and IMR-32 cell lines.

## 4. Conclusions

Preliminary insights towards the structure-activity relationships among a series of xanthone derivatives are proposed. The xanthone derivatives bearing substituent groups such as diprenyl, dipyrano and prenyl-pyrano displayed cytotoxicity towards almost all the tested cancer cell lines.
